# Octogenarian With Acromion Stress Fracture Nonunion With Reverse Total Shoulder Prosthesis Reconstructed With Plates and Screws: A Case Report

**DOI:** 10.7759/cureus.42865

**Published:** 2023-08-02

**Authors:** Jamison K Walker, Brett W Richards, John T Cronin, John G Skedros

**Affiliations:** 1 School of Medicine, Campbell University School of Osteopathic Medicine, Buies Creek, USA; 2 Shoulder & Elbow, Utah Orthopaedic Specialists, Salt Lake City, USA; 3 Orthopaedics, University of Utah, Salt Lake City, USA

**Keywords:** non-union, open reduction internal fixation, reverse total shoulder arthroplasty, acromion, stress fracture

## Abstract

An 85-year-old male underwent open reduction and internal fixation (ORIF) for a displaced acromion stress fracture that occurred two years prior. The complete fracture occurred two months after an ipsilateral reverse total shoulder arthroplasty (RTSA). Four weeks after his RTSA, the pain was felt at the posterior-superior shoulder with activities of his daily living as a rancher, reflecting non-compliant use. A stress fracture was suspected but not detected radiographically. Four weeks later, higher pain occurred after he lifted a hay bale, and a displaced basilar acromion fracture was detected. Non-operative management spanned 20 months, which he ultimately deemed unacceptable because of pain with minor activities. ORIF was then done. Approximately 10 months after the ORIF surgery, both plates sustained fatigue breakage; however, the fracture consolidated, and his pain remained low. He is the oldest patient described to ultimately have a successful acromion fracture ORIF and only the third reported acromion fracture ORIF in octogenarians following RTSA. We report the ORIF technique, its good outcome, and a literature review of elderly patients who had ORIF for this problem.

## Introduction

Acromion and scapular spine fractures are rare but potentially severe complications arise following a reverse total shoulder arthroplasty (RTSA). Mahendraraj et al. [1] retrospectively reviewed 6,755 patients who underwent an RTSA and found that 200 (3.0%) sustained acromion fractures and 64 (0.9%) sustained scapular spine fractures. Additional studies show that the frequency of acromion and scapular spine fractures following RTSA ranges from 0.8% to 11.2% [2-7]. Some of these are considered stress fractures or are caused by minor trauma; however, more significant trauma occurs in over one-half of cases [4, 8-11]. In general, acromion and scapular spine fractures are classified as either acute traumatic or chronic stress fractures. Stress fractures following RTSA are often associated with osteoporosis seen with aging [5, 7, 12-14].

Although acromion fractures that occur in the setting of an RTSA can lead to poor outcomes [2, 8-10], treatment is often conservative, especially for non-displaced or minimally displaced fractures. There are two popular classifications for acromion and scapular spine fractures [15-16]. Levy et al. [15] reported that type I and II acromion fractures occur at a nearly equal rate; type III fractures are the least common [7, 17-19]. The 85-year-old patient reported here had a displaced Levy type II fracture, which is near the base of the acromion (type I is nearest to the more distal aspect of the acromion; type III is where the acromion merges with the scapular spine). Our patient’s fracture occurred several weeks after having an RTSA on the same side.

Most stress fractures of the acromion and scapular spine that occur in patients with advanced age are treated non-operatively because of their lower usage of the shoulder. Our patient, however, demanded greater use of his injured shoulder than was provided after 20 months of non-operative management. The most novel aspects of our case are: (1) our patient was a high-functioning 85-year-old at the time of his acromion fracture fixation surgery, and (2) he had a good result with reconstruction with orthogonal double plates, screws, and bone grafting, despite fatigue breakage of both plates. We also review the literature reporting outcomes of surgically and non-surgically treated acromion and scapular spine fractures in septuagenarian and octogenarian patients who had an RTSA on the same side.

## Case presentation

Our 83-year-old (173 cm tall, 85 kg weight; body mass index, BMI = 28.5) right-hand dominant male patient underwent a right-side RTSA because of shoulder pain and weakness caused by a large rotator cuff tear and glenohumeral arthritis. He did not drink or smoke, but he had hypertension that was controlled with medication and had a cardiac stent placed six years prior for coronary artery disease. For this latter problem he took warfarin daily. His American Society of Anesthesiologists (ASA) score (i.e., physical status classification system) was ASA III (one or more severe systemic diseases).

The RTSA was done by one of us (JGS) and without immediate perioperative complications (prosthesis: Delta XTEND(TM), Depuy, Warsaw, IN; 12 mm stem, cementless: 9 mm standard polyethylene liner; standard baseplate, 42 diameter glenosphere with 2 mm lateral offset). The patient experienced pain over the posterior acromion four weeks after surgery. There was no specific traumatic event and he reported “minimal shoulder use.” He later admitted to non-compliance with our post-operative protocol for our elderly patients, which includes: (1) no lifting for four weeks, (2) only active-assisted motion over shoulder level between three and eight weeks, (3) lifting up to one pound (0.45 kg) from week four to eight, and (4) lifting up to 10 lbs (4.5 kg) after week 12 but never repetitively over shoulder level. Consequently, we considered this pain as early evidence of an acromion stress fracture and not a perioperative complication. However, the differential diagnosis in this case includes other occult fracture or musculotendinous strain in the vicinity of the prosthetic components, especially near the glenosphere. In these cases, we obtain three-view radiographs (true anterior-posterior, scapular Y, and axillary lateral). These radiographs did not show a fracture, and all of the RTSA components were without obvious complication. The patient was advised to reduce use of his shoulder, avoid formal physical therapy, and return for radiographs in six weeks.

**Figure 1:**
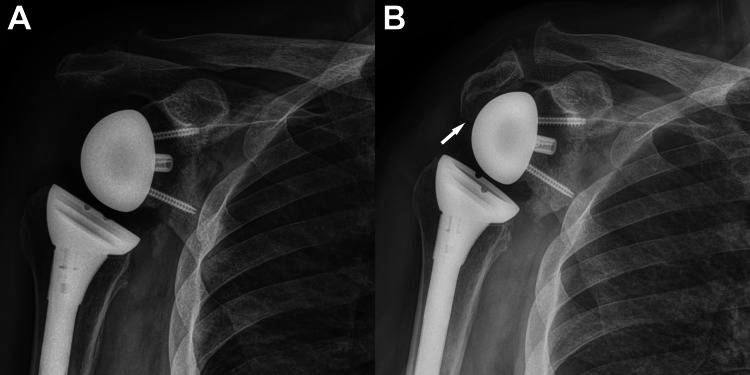
Anterior-posterior radiographs of right RTSA. A. Our patient’s RTSA at two months (March 2020) after implantation surgery (before the acromion fracture). B. His RTSA showing the acromion fracture (November 2021); note the narrowed subacromial space (arrow). RTSA, reverse total shoulder arthroplasty

**Figure 2:**
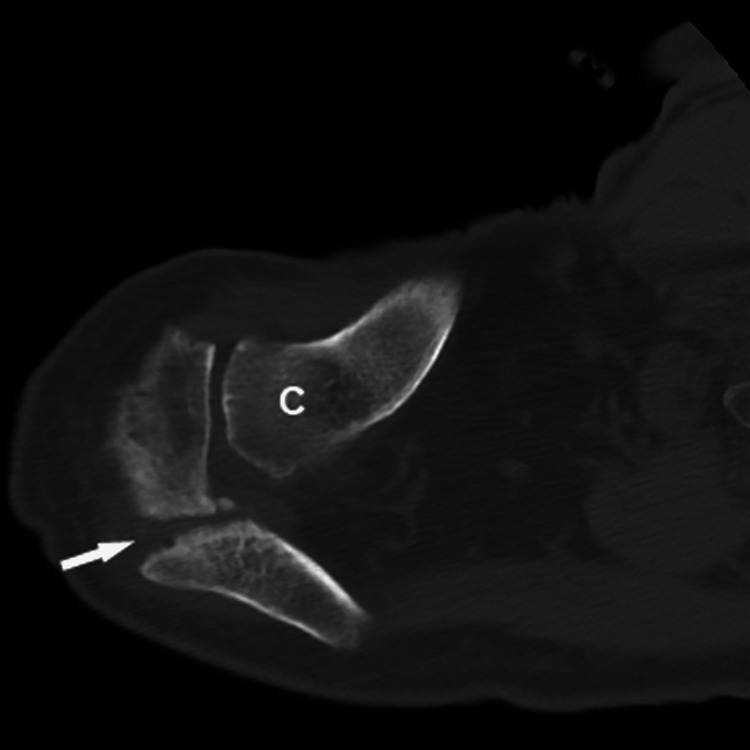
Axial CT scan of the right shoulder. Acromion fracture is indicated by the arrow; C, clavicle

Six weeks later the patient reported increased pain following lifting a bale of hay (approximately 27 kg). He informed us that he was significantly more active than previously reported. Radiographs and CT scan images showed a displaced Levy type II acromion fracture (Figures 1-2). CT imaging also revealed a fracture along the posterior-inferior aspect of the glenoid near a fixation screw, which we did not consider to be a stress fracture in the typical context of this diagnosis (i.e., this occurred from a discrete lifting event, not from repetitive use as was the case for the acromion fracture). Conservative treatment was advised due to the patient’s advanced age and presumably age- and warfarin-related reduction in bone mass and/or quality. There were no other comorbidities that contributed to this treatment plan. However, the patient did not have a work-up for poor bone-health (vitamin D deficiency, osteoporosis, etc.).

The patient was sent to physical therapy for gentle stretching and isometric strengthening, which provided modest improvement. He returned for revaluation multiple times over the next 1.5 years and expressed his desire for fracture repair stating that he ‘couldn’t live with the pain, lack of shoulder mobility, and decreased daily activity.’ He stated that even putting on a shirt was moderately painful. We eventually referred him to another shoulder surgeon specialist for a second opinion regarding performing an ORIF of the acromion fracture (the glenoid fracture had healed). He recommended conservative treatment because of the patient’s advanced age. The patient returned to our clinic insistent on having ORIF so that he could resume some greater use of the shoulder because he was certain that this would enhance his quality of life. We agreed to do this after he reassured us that he would curtail some of his prior shoulder activities.

Open reduction and internal fixation was performed 20 months following the acromion fracture, which was nearly two years following the RTSA. At that time, the patient was 85 years old, and he had the same ASA score that he had two years prior (ASA III). Surgery was done with the patient in a semi beach-chair position. The nonunion site was 100% displaced, angulated inferiorly about 20°, and gapped 15 mm. The fibrous tissue at the nonunion site was avascular and was excised, and the medial and lateral metaphyseal bone was curetted to create 3-4 mm depressions (to increase surface area for bone graft). A bur was used to create bleeding bone along the cortical margins and also 2 cm along the superior and inferior aspects of the acromion near the nonunion site. A mixture of fresh-frozen cancellous bone allograft, demineralized bone allograft, and recombinant bone morphogenetic protein was placed within and around the prepared fracture site. More specifically, this included:

1. 10 mL of cancellous “crouton” allograft that was manually crushed with a rongeur with 2 mL of surgical-site blood and 2 mL of demineralized human bone matrix in gel (GraftonTM Gel Medtronic, Minneapolis, MN). GraftonTM gel is demineralized cancellous bone derived from human cadavers. 

2. A small Infuse® bone graft kit (2.8 cc) containing two sterile absorbable sponges (2.5 cm x 5 cm) soaked with fluid containing human bone morphogenetic protein-2 (sterile rhBMP-2, 4.2 mg) (Medtronic Sofamor Danek USA, Inc., Memphis, TN). The sponges were cut into strips of about 2 mm by 10 mm. The Infuse® bone graft kit aims to stimulate natural bone formation.

The lateral acromion was elevated and reduced so that the remaining bone surfaces were in direct contact, which made the region about 5 mm shorter than the normal prior anatomy. 

**Figure 3:**
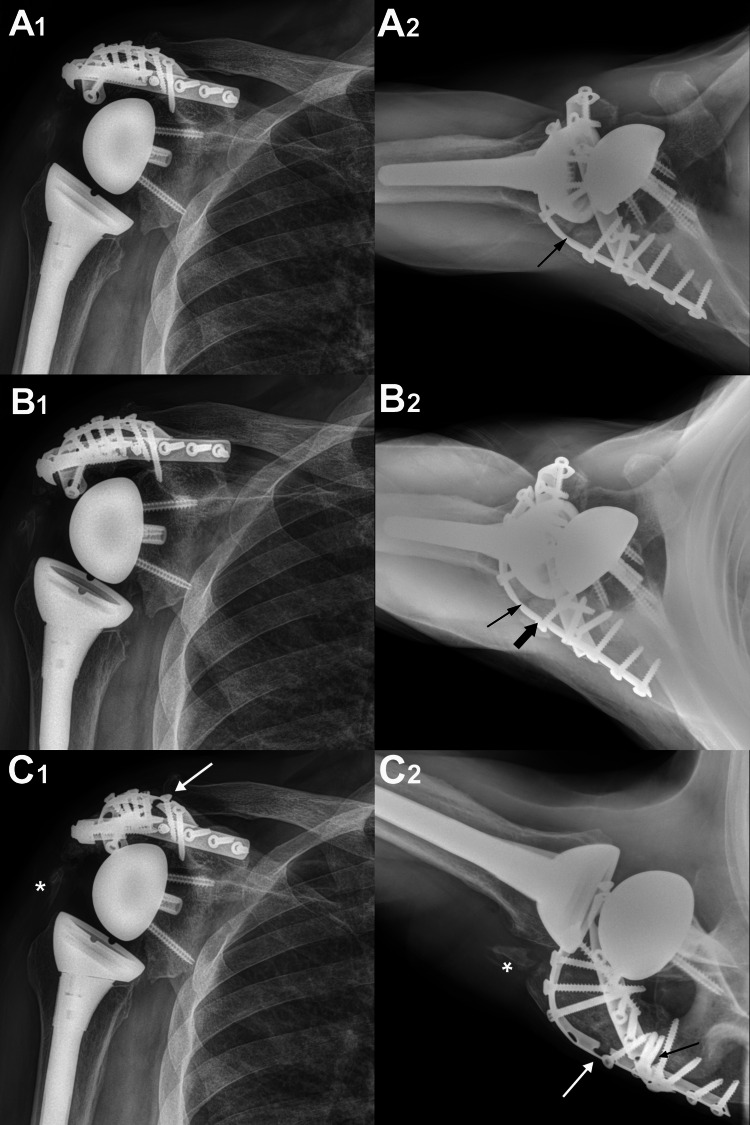
Our patient’s RTSA with double plating of the acromion fracture. Our patient’s RTSA with double plating of the acromion fracture in anterior-posterior and axillary lateral views at four months after the fracture reconstruction (A1 and A2), and at 11 months after fracture reconstruction (B1 and B2). The thin arrows shown in the axillary lateral views of A2 and B2 point to the grafted nonunion site. The thicker shorter arrow in B2 points to the first evidence of plate breakage.  Progressive calcification had occurred over this time interval (4-18-month follow-up period). The 18-month post-ORIF radiographs in part C show that fatigue fractures of the plates are now obvious (one arrow in C1; two arrows in C2), which are presumably from cyclic loading during his activities of daily living as a rancher. The nonunion subsequently consolidated (C2, beneath the plate hole without a screw). There was also mature-appearing heterotopic ossification in the upper-lateral deltoid (asterisks). RTSA, reverse total shoulder arthroplasty

As described by Bauer et al. [5], double plating of the acromion was performed by placing two 1/3 tubular 10-hole plates 90⁰ to each other; one was cut to allow for enhanced proximity to the bone (hence one plate had nine holes). The plates we used were non-locking and were contoured to fit across the superior and posterior-lateral aspects of the acromion and scapular spine. In total, 16 screws were used (Figure 3A). To relieve some tension in the deltoid, a large abduction pillow was used for six weeks, followed by a small abduction pillow for an additional three weeks. We followed a slow/conservative post-operative protocol used after open repair of large/massive rotator cuff tears [20].

**Figure 4:**
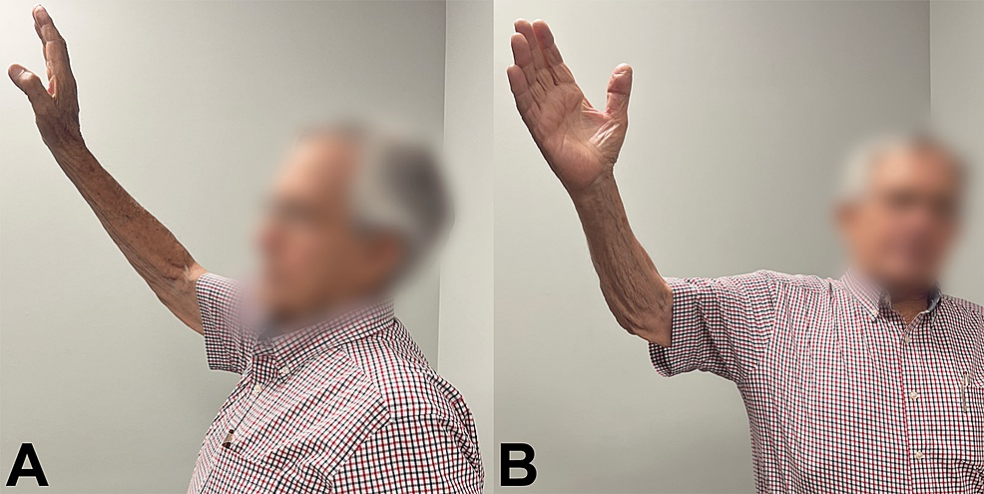
ROM at 18-month follow-up. Shoulder motion at final follow-up 18 months after fracture reconstruction. The patient’s active flexion was 110°, abduction was 90°, external rotation was 50°, and internal rotation to L5. ROM, range of motion

**Figure 5:**
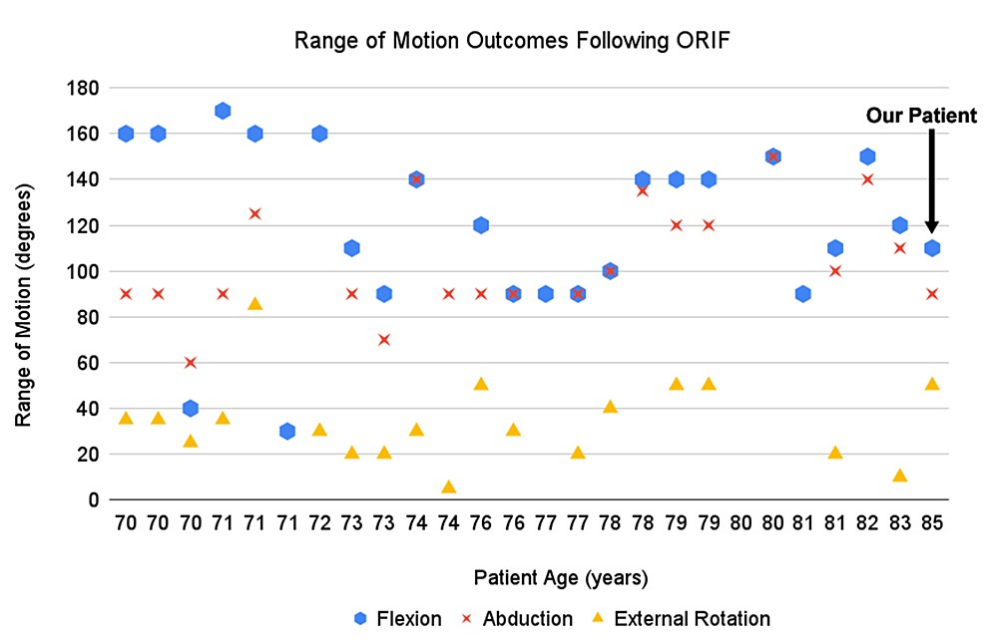
ROM outcomes following ORIF. Data for this figure was taken from the references in Tables 1-2. ROM, range of motion; ORIF, open reduction and internal fixation

**Figure 6:**
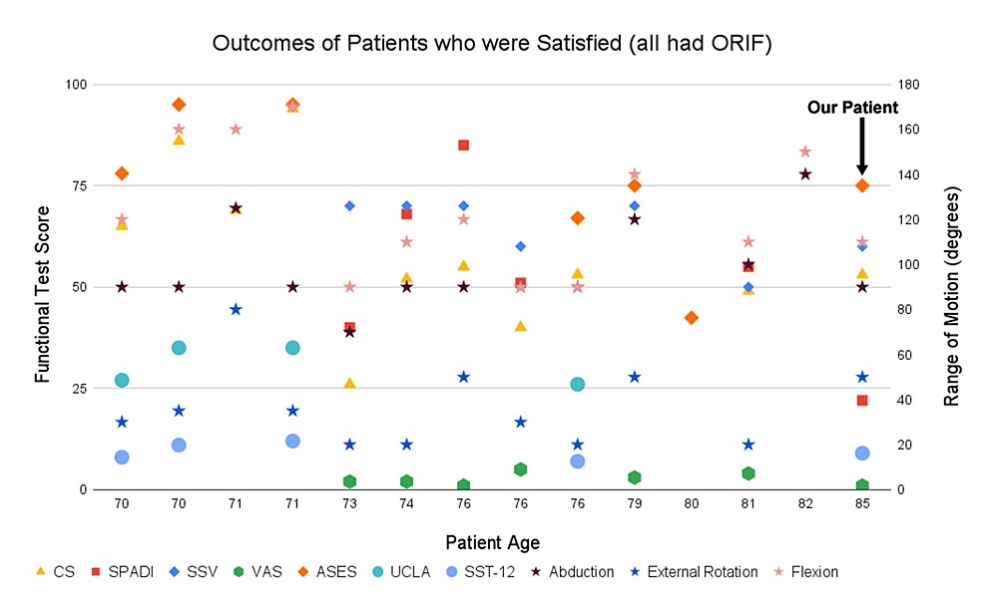
Outcomes of patients who were satisfied (all had ORIF). Range of motion (ROM) is in degrees (second y-axis); CS, constant score; SPADI, Shoulder Pain and Disability Index; SSV, subjective shoulder value; VAS, visual analog scale (10 cm); ASES, American Shoulder and Elbow Surgeons; UCLA, University of California-Los Angeles shoulder score; SST-12, 12-item simple shoulder test; ROM including flexion, abduction, and external rotation are reported on the right axis while functional test outcomes are reported on the left axis. See footnote of Table 3 for details of outcome scoring methods. ORIF, open reduction and internal fixation

The patient was followed for 18 months at the time of this report. He was 86 years old at that time. He reported being satisfied with his shoulder function and stated that he was happy he underwent the ORIF surgery. His active flexion was 110°, abduction was 90°, external rotation was 50°, and internal rotation to L5 (Figure 4). These ranges of motion were the same at the 11-month follow up (he was 85 years old) and 18-month follow up (he was 86 years old). He felt comfortable performing various low-intensity tasks at his farm, such as feeding cattle and light shoveling. There was no pain over the site of the prior lucency at the site of bone grafting (Figure 3B2), but “tolerable pain” (1-2/10 on a VAS) with activity was noted over the superior-lateral deltoid where some heterotopic ossification (HO) had developed (Figure 3C2). Given the patient’s overall satisfaction, no further surgery was considered for the HO. Post-operative radiographs at 18 months after surgery are shown in Figure­ 3C. Notably, both plates had broken (Figure 3C3), and the first evidence of this was at his 11-month follow-up visit (Figure 3B2). We considered these plate breakages to be the result of cyclic fatigue failure because he did not experience a discrete traumatic event or any significantly increased acute pain. Radiographs showed that minor angulation resulted at the fracture site and healing had progressed where lucency (delayed union) had been seen at the 11-month post-operative visit (compare Figure 3 images B2 with C2). His 18-month post-operative outcome measures are listed in Tables 1-3 and Figures 5-6 in the context of findings of our literature review (described below).

**Table 1:** Outcomes of acromion/scapular spine fractures treated with ORIF in patients ≥ 80 years old. Range of motion (ROM) is in degrees; SSV, subjective shoulder value (100 is completely normal shoulder); CS, constant score; ASES, American Shoulder and Elbow Surgeons; UCLA, University of California-Los Angeles shoulder score; SST-12, 12-item simple shoulder test; SPADI, shoulder pain and disability index (0-100, 100 is best); VAS, 0-10 visual analog scale (10 is worst pain possible); ABT ST, abduction strength. See footnote of Table 3 for details of outcome scoring methods.

Study	Final follow up (months)	Age	Treatment modality	Outcomes	ROM
Debeer ­and Robyns 2005 [40]	2	83	Single plate - non-locking 1/3 tubular plate	Pain-free regained the same active and passive motion as before the fall	Flexion 120; abduction 110; external rotation 10; internal rotation L3
Wahlquist et al. 2011 [36]	12	82	Lag screw and tension band	Satisfied, fracture union	Flexion 150; abduction 140
Hess et al. 2018 [41] (this patient is also reported by Stückelberger et al. 2020 [43])	12	80	Single plate - modified cruciform pilon plate	Satisfaction not reported, fracture union; CS 71; CS opposite side 81; SSV 90	Flexion 150; abduction 150
Neyton et al. 2019 [18]	80	81	Double plates - perpendicular AO plates	Satisfaction not reported; nonunion; pain score 5/15; CS 27	Flexion 90
Toft and Moro 2019 [25]	12	81	Double plates - one large plate superior [a locking compression plate (LCP) distal humerus, or variable angle (VA)-LCP olecranon; or VA-LCP distal humerus plate (lateral)] and one small plate (quarter tubular plate) inferior scapular spine	Satisfied; CS 49; SPADI 55; SSV 50; VAS 4; ABD ST 2.0	Flexion 110; abduction 100; external rotation 20; internal rotation L3
Konstantinidis et al. 2020 [42]	48	80	Single plate - 3.5 mm pelvic reconstruction plate	Satisfied; fracture union; ASES 42; SF36 45	No ROM data
Current case 2022	18	85	Double plates - 1/3 tubular plates	Both plates broke but he was satisfied with the outcome, calcifying union; CS 53; SST-12 9; VAS 1; SPADI 22; SSV 60; ASES 75	Flexion 110; abduction 90; external rotation 50; internal rotation L5

**Table 2:** Outcomes of acromion/scapular spine fractures treated with ORIF in patients 70-79 years old. Range of motion (ROM) is in degrees; SSV, subjective shoulder value (100 is completely normal shoulder); CS, constant score; ASES, American Shoulder and Elbow Surgeons; UCLA, University of California-Los Angeles shoulder score; SST-12, 12-item simple shoulder test; SPADI, Shoulder Pain and Disability Index (0-100, 100 is best); VAS = 0-10 visual analog scale (10 is worst pain possible); ABT ST, abduction strength; DASH (and QuickDASH), disabilities of the arm, shoulder, and hand questionnaire. See the footnote of Table 3 for details of outcome scoring methods.

Study	Final follow up	Age	Treatment modality	Outcomes	ROM
Wahlquist et al. 2011 [36]	12 months	70	Double plates - 3.5 mm plate superiorly and 2.7 mm plate posteriorly	Satisfaction not reported; fracture union	Flexion 40; abduction 60; external rotation 25; internal rotation gluteal
Rouleau and Gaudelli 2013 [44]	18 months	71	Double plates - 90° 3.5mm Synthes LCP plates, first superiorly and the second posteriorly	Satisfied; QuickDASH 29.5, CS 69; CS opposite shoulder 85	Flexion 160; abduction 125; external rotation 85; internal rotation 60
Camarda et al. 2015 [27]	12 months	78	Two Synthes AO mesh plates were fashioned to create hooks for the medial scapula and the acromion	Satisfaction not reported; fracture united; ASES 52; DASH 48; OSS 28; pain-free	Flexion 100; abduction 100; external rotation 40
Lópiz et al. 2015 [2]	24 months after the fracture	74	Not stated	Satisfaction not reported; CS 60	Abduction 90; external rotation 5
Kennon et al. 2017 [32]	36 months	77	Not stated	Satisfaction not reported; ASES 67; UCLA 26; SST-12 7; CS 53; pain-free	Flexion 90; abduction 90; external rotation 20; internal rotation back pocket
Kennon et al. 2017 [32]	36 months	70	Not stated	Satisfaction not reported; ASES 78; UCLA 27; SST-12 8; CS 65; pain-free	Flexion 120; abduction 90; external rotation 30; internal rotation back pocket
Kennon et al. 2017 [32]	48 months	71	Not stated	Satisfaction not reported; ASES 95; UCLA 35; SST-12 11; CS 94; pain-free	Flexion 170; abduction 90; external rotation 35; internal rotation T10
Kennon et al. 2017 [32]	24 months	70	Not stated	Satisfaction not reported; ASES 95; UCLA 35; SST-12 11; CS 86; pain-free	Flexion 160; abduction 90; external rotation 35; internal rotation back pocket
Hess et al. 2018 [41] (This patient is also reported by Stückelberger et al. 2020 [43])	12 months	78	Single plate - modified cruciform pilon plate	Satisfaction not reported; fracture union; CS 55; CS opposite side 79; SSV 70	Flexion 140; abduction 135
Toft and Moro 2019 [25]	17 months	76	Double plates - one large plate superior (see the Toft and Moro patient in the previous table) and one small plate (quarter tubular plate) inferior to the scapular spine	Satisfied; CS 55; SPADI 75; SSV 70; VAS 1; ABD ST 1.5	Flexion 120; abduction 90; external rotation 50; internal rotation T7
Toft and Moro 2019 [25]	12 months	76	Double plates - one large plate superior; one small plate (quarter tubular plate) inferior to the scapular spine	Satisfied; CS 40; SPADI 51; SSV 60; VAS 5; ABD ST 1.5	Forward flexion 90; abduction 90; external rotation 30; internal rotation sacral
Toft and Moro 2019 [25]	14 months	74	Double plates - one large plate superior; one small plate (quarter tubular plate) inferior to the scapular spine	Satisfied; CS 52; SPADI 68; SSV 70; VAS 2; ABD ST 2.4	Flexion 110; abduction 90; external rotation 20; internal rotation L1
Toft and Moro 2019 [25]	12 months	73	Double plates - one large plate superior; one small plate (quarter tubular plate) inferior to the scapular spine	Satisfied; CS 26; SPADI 40; SSV 50; VAS 3	Flexion 90; abduction 70; external rotation 20; internal rotation gluteal
Neyton et al. 2019 [18]	87 months	77	Double plates - two perpendicular AO plates	Satisfaction not reported; fracture union; pain score 5/15; CS 28	Flexion 90
Bauer et al. 2020 [5]	12 months	74	Double plates - 90° 1/3 tubular plate [lateral clavicular plate (Synthes/LCP)] double plate construct 3.7/2.7 mm plate superiorly and 2.4 mm posteriorly	Satisfaction not reported; fracture union; SSV 80%; CS 67; pain-free	Flexion 140; abduction 140; external rotation arm at side 30; internal rotation L5
Tashjian et al. 2020 [45]	Unclear (minimum of 2 years from RTSA)	71	Double plates (perpendicular AO type, small fragment)	Satisfaction not reported; fracture union; ASES 25; VAS 5	Flexion 30 (no other ROM details)
Kim et al. 2021 [46]	24 months	79 (two shoulders)	Single plate - pre-contoured acumed distal clavicle plate	(Both shoulders) satisfied; VAS 3; ASES 75% SSV 70%	(Both shoulders) Flexion 140; abduction 120; external rotation 50; internal rotation L3
Khwaja et al. 2021 [47]	12 months	72	Single plate - clavicle plate	Satisfaction not reported; patient returned to baseline function; fracture union; pain-free	(Returned to baseline) Flexion 160; external rotation 30; internal rotation L2

**Table 3:** Review of individual patient satisfaction and outcomes at final follow up (all had ORIF). Range of motion (ROM) (flexion, abduction, external rotation, and internal rotation) are in degrees. CS, constant score [100-point system based on four categories: pain (15 pts.), activities of daily living (20 pts.), strength (25 pts.), and ROM (40 pts.)]; SPADI, Shoulder Pain and Disability Index (100-point system based on two categories weighted equally: pain and patient disability; a score of 0 is best and a score of 100 is worst. A higher score shows greater disability); SSV, subjective shoulder value (100-point system based on the subjective patient evaluation); VAS, 0-10 visual analog scale (10-point system based on patient subjective pain, 0 being no pain and 10 being the worst pain possible); ASES, American Shoulder and Elbow Surgeons [100-point system based on two categories: pain (50 pts.) and activities of daily living (50 pts.)], UCLA, University of California-Los Angeles shoulder score (35-point system that includes evaluation of five categories: pain (10 pts.), active forward flexion (5 pts.), satisfaction of patient (5 pts.), activities of daily living (10 pts.), and strength of forward flexion (5 pts.); a score of ≥ 27 reflects a good/excellent score), SST-12, 12-item simple shoulder test (series of 12 yes or no questions based on quality of life).

Study	Age	CS	SPADI	SSV	VAS	ASES	UCLA	SST-12	Flexion	Abduction	External rotation	Internal rotation
Wahlquist et al. 2011 [36]	82	-	-	-	-	-	-	-	150	140	-	-
Rouleau and Gaudelli 2013 [44]	71	69	-	-	-	-	-	-	160	125	80	60
Kennon et al. 2017 [32]	77	53	-	-	-	67	26	7	90	90	20	Gluteal
Kennon et al. 2017 [32]	70	65	-	-	-	78	27	8	12	90	30	Gluteal
Kennon et al. 2017 [32]	71	94	-	-	-	95	35	12	170	90	35	T10
Kennon et al. 2017 [32]	70	86	-	-	-	95	35	11	160	90	35	Gluteal
Toft and Moro 2019 [25]	81	49	55	50	4	-	-	-	110	100	20	L3
Toft and Moro 2019 [25]	76	55	85	70	1	-	-	-	120	90	50	T7
Toft and Moro 2019 [25]	76	40	51	60	5	-	-	-	90	90	30	Sacral
Toft and Moro 2019 [25]	74	52	68	70	2	-	-	-	110	90	20	L1
Toft and Moro 2019 [25]	73	26	40	70	2	-	-	-	90	70	20	Gluteal
Konstantinidis et al. 2020 [42]	80	-	-	-	-	42	-	-	-	-	-	-
Kim et al. 2021 [46]	79	-	-	70	3	75	-	-	140	120	50	L3
Our patient	85	53	22	60	1	75	-	9	110	90	50	L5

## Discussion

Reverse total shoulder arthroplasties (RTSAs) comprise approximately one-third to one-half of all shoulder arthroplasties performed yearly in the United States [4]. As the number of RTSAs increases, surgeons can expect to see complications with greater regularity. Although acromion and scapular spine fractures are well-known complications of RTSAs, there is no consensus regarding their ideal treatment in this context [4-7, 16, 21-24]. Acromion and scapular spine fractures following a RTSA present unique challenges because of the high malunion or nonunion rates, decreased functional outcomes, and variable results of ORIF [4, 7, 21, 24-25]. Conservative treatment with sling immobilization is often the first choice for management; surgical intervention is usually reserved for malunions, nonunions, or acute displaced fractures [7, 26-27]. Because of our patient’s advanced age (and presumed relatively lower shoulder use), we initially avoided surgery even though the acromion fracture was significantly displaced.

To highlight some of the difficulties of treating acromion and scapular spine fractures following RTSA, Neyton et al. [18] retrospectively reviewed ~1,000 RTSAs. They found that of the 16 individuals who sustained an acromion or scapular spine fracture (all were treated non-operatively), only 40% and 33%, respectively, resulted in adequate healing. In a review of 3,838 RTSAs, Patterson et al. [4] reported that there were 159 ipsilateral acromial fractures (4.1%) of which 20 were treated operatively. Fifty-five of the 139 non-operatively treated acromion fractures reported by Patterson et al. [4] came from two studies. In those two studies, union rates ranged from 50% to 60% [2, 28]. Hence, non-operative treatment resulted in higher nonunion rates. Despite having lower nonunion and complication rates, ORIF was not shown to be clinically superior. 

Our literature review revealed 87 patients who could be clearly identified as being ≥70 years-old and had an acromion or scapular spine fracture after a RTSA on the same side [2, 5, 8-9, 18, 25-47]. There are other published studies that, although they identify patients with acromion and scapular spine fractures after RTSA, report only average age. For example, Mahendraraj et al. [1] reported on 200 acromion fractures and 64 scapular spine fractures, but only reported 72.7 ± 7.7 years for ages of patients with acromion fractures. Some studies also do not report post-operative outcomes. These are the main reasons why we did not include some studies in our literature review.

Sixty-three of the 87 patients (again, all were ≥70 years of age) that we identified were treated non-operatively for acromion or scapular spine stress fractures that were on the same side of their RTSA [2, 8-9, 18, 26, 28-39]. Of these 63 conservatively treated patients, 16 went to union, 17 resulted in nonunion, three were malunions, and in 27 the extent of healing was not reported. Of these 87 patients, 24 (28%) had ORIF for their acromion or scapular spine fracture [2, 5, 18, 25, 27, 32, 36, 40-48]. Six of the 24 ORIF cases were in patients that were ≥80 years old, with the oldest being 83 years (Table 1) [18, 25, 36, 40-43]. Of these six octogenarian patients (which does not include our patient), only two underwent ORIF with double plates. Three of the other four patients underwent ORIF with a single plate; the fourth patient had a tension-band construct. These four patients had higher range of motion and outcome measures, but double-plated patients were satisfied with their shoulder despite having lower scores. Additionally, the octogenarian patients with the single plate or tension band had higher range of motion (ROM) in flexion (120°-150°) and abduction (110°-140°) versus the double-plate flexion (90°-110°) and abduction (100°). Single- and double-plate patients had similar internal and external rotations.

Of the ≥70 years old patients who underwent an ORIF for acromion or scapular spine fracture following RTSAs, 10 were reported to have union; one was reported as nonunion, and healing was not reported in 13 fractures (Tables 1-2) [2, 5, 18, 25, 27, 32, 36, 41, 43-47]. Our patient’s fracture site showed good healing on radiographs taken at 18 months after the ORIF surgery despite having both plates sustain fatigue fracture.

No age-related recommendations are stated in any of the published studies we reviewed regarding when and how to perform ORIF for acromion or scapular spine fractures in the setting of an ipsilateral RTSA. Toft and Moro (2019) reported a series of five female patients aged 73, 73, 76, 76, and 81 who sustained scapular spine fractures and had subsequent ORIF. They found ORIF to be beneficial in all of these patients, and they all stated that they would undergo the ORIF again. Although the authors did not report fracture healing due to a lack of affirmative post-ORIF CT imaging, they felt that the patients’ satisfactory results implied union or at least a stable, painless pseudoarthrosis. Although these results are encouraging, the small sample size limits the general applicability of their findings to all patients ≥70 years of age.

Many studies that we reviewed had different methods of determining post-operative outcomes (Tables 1-3). Post-operative range of motion in the 70-79 age group at final follow up was: (1) forward flexion ranged from 30° to 170° with an average of 115°, (2) abduction ranged from 60° to 140° with an average of 98°, and (3) external rotation ranged from 5° to 85° with an average of 35°. Post-operative ROM in the >80 age group was similar in some planes: (1) forward flexion ranged from 90° to 150° with an average of 124°, (2) abduction ranged from 100° to 150° with an average of 125°, and (3) external rotation was only reported in two patients at 10° and 20° (Figures 5-6).

There are three final aspects of our patient’s case that warrant some consideration, the second being especially important for surgeons who are faced with a similar problem in an elderly patient. First, when considering the etiology of the two fractures in our elderly patient (near-basilar acromion and glenoid base), we think that they were caused at different times by different activities: (1) the acromion fracture occurring from the excessive daily shoulder motion, including reaching upward, without allowing for adequate time to heal the nascent acromion fracture, and (2) the glenoid fracture from stress concentration at the inferior glenoid screw from the discrete event when he lifted a hay bale. Second, even though both of the one-third tubular plates broke in our patient, his pain level did not increase and his activity level did not decrease. This is because the fracture appeared to show progressive healing, likely because of improved contact at the prior delayed union site after plate breakage. We used two one-third tubular plates with orthogonal placement because of their low profile and the high likelihood that healing would progress because of the anticipated/expected low-level physical activity in our octogenarian patient [5]. However, our patient was more active post-operatively than was recommended. In retrospect, stacked one-third tubular plates [49] or conventional/locking reconstruction plates would have been a better choice in this case [50], the latter being commonly used in septuagenarians and octogenarians with acromion/scapula fractures in the setting of a prior RTSA (Tables 1-2). Third, evaluating our patient for poor bone health prior to his index RTSA may have suggested that he was at higher risk for the acromion fracture he later sustained [51]. Had this been the case, perhaps, we would have been more emphatic on reduced use of the shoulder during the first several months after surgery. Additionally, hip fracture literature suggests that vitamin D deficiency also plays a role, alongside osteoporosis, in increasing the risk of hip fragility fractures [52-53]. More studies are needed to explore the influence of osteoporosis and vitamin D deficiency in elderly patients who suffer post-RTSA acromion stress fractures.

## Conclusions

The 85-year-old patient reported here is the oldest that we could locate who had a successful ORIF of an acromion fracture and only the third report of double-plating of an acromion fracture in octogenarians following an ipsilateral RTSA. Notably, both one-third tubular plates had fatigue-related breakage at approximately 10 months after his ORIF surgery. Nevertheless, he showed progressive healing and had an acceptable result at 18 months after the ORIF surgery, and no additional surgery was anticipated.
